# Advanced Testing Protocols Simulate Failures and Validate Antioxidant Polyethylene in Ankle Implants

**DOI:** 10.1002/jor.26103

**Published:** 2025-05-19

**Authors:** Ryan Siskey, Patrick Hall, Ruth Heckler, David Safranski, James Johnson, Ariel Palanca

**Affiliations:** ^1^ Exponent Inc. Philadelphia Pennsylvania USA; ^2^ School of Biomedical Engineering, Science, and Health Systems Drexel University Philadelphia Pennysylvania USA; ^3^ Surgical Division Enovis Austin Texas USA; ^4^ Foot and Ankle Division Enovis Atlanta Georgia USA; ^5^ Palomar Health Medical Group San Diego California USA

**Keywords:** oxidation and antioxidant effects, polyethylene fatigue resistance, total ankle replacement (TAR), varus‐valgus rotation, vitamin E‐stabilized polyethylene

## Abstract

Total ankle replacement (TAR) has become an effective treatment for end‐stage ankle osteoarthritis. Multiple factors, including patient characteristics, surgical technique, alignment, and bearing surfaces, influence TAR survivorship. Polyethylene (PE) fatigue is a key consideration in improving outcomes. This study establishes a novel, clinically relevant testing protocol incorporating varus‐valgus rotation to simulate polyethylene fatigue failures observed in mobile‐bearing total ankle replacements. Using this robust methodology, we evaluated the impact of oxidation and antioxidant stabilization on ultrahigh‐molecular‐weight polyethylene (UHMWPE) performance in a mobile bearing implant application. A six‐degree‐of‐freedom simulator was used to iteratively adjust loading parameters (1500–3000 N, −4° to +8° flexion‐extension, ±5° axial rotation, and ±3° or ±8° varus‐valgus rotation at 37 ± 3°C in 20 g/L bovine serum) until clinically observed midline fractures were replicated. Oxidation levels were measured by Fourier‐transform infrared spectroscopy per ASTM F2102. This validated loading protocol was then applied to conventional (25 kGy GUR 1020) and vitamin E‐stabilized (75 kGy GUR 1020‐E) UHMWPE inserts and tested to visible fracture or a 3‐million‐cycle runout. Post‐test fractographic analysis identified crack initiation sites. Conventional aged UHMWPE demonstrated fatigue failure under varus‐valgus rotation (OI = 2.59 ± 1.11) but no failure without rotation. Vitamin E‐stabilized UHMWPE showed no fatigue failure after 3 million cycles, even under varus‐valgus rotation (OI = 0.23 ± 0.02). Fractography revealed fractures originating at the trough and propagating with cyclic loading. Oxidation significantly reduces polyethylene fatigue life, and varus‐valgus rotation exacerbates this effect in mobile bearing TAR implants. Antioxidant‐stabilized UHMWPE showed promising resistance to fatigue and oxidation. These findings support the role of antioxidant stabilization in improving TAR performance, and the protocols developed here provide a framework for assessing the safety of alternative materials.

## 1 Introduction

Total ankle replacement (TAR) has become an effective means of treating end‐stage ankle osteoarthritis [1]. However, recent registry data have reported up to a 30.5% [2, 3] all‐cause revision rate after total ankle arthroplasty, highlighting how multiple factors—including patient‐specific variables, surgical technique, coronal plane alignment, and implant bearing surface materials [4]—can influence TAR survivorship. Currently, there are numerous FDA‐approved/cleared TAR implant devices available; all use various formulations of ultra‐high‐molecular‐weight polyethylene (UHMWPE) as the inlay bearing surface [5], as do many other total joint replacement (TJR) devices. Although bearing surface stresses vary according to implant design and material properties, UHMWPE fractures have been reported in various TJR devices [6, 7, 8, 9, 10, 11]. These failures, in combination with the increasing number of joint replacement surgeries in ever younger patients and increased life expectancy of older patients, demand maximizing of UHMWPE fatigue resistance as a means of bolstering implant lifespan and improving TAR outcomes.

While reports in TAR are limited, failure of UHMWPE components in TJR is generally attributed to the polymer microstructural properties or oxidation (e.g., molecular weight, fusion defects, crystallinity, or crosslinking) [11, 12, 13, 14]. Several material processing treatments such as gamma irradiation crosslinking (to increase crosslinking density; measured in units of ionizing radiation, kGy) and annealing (to reduce free radicals) have successfully improved wear and oxidation resistance in orthopedic UHMWPE. Although crosslinked polyethylene has achieved favorable long‐term clinical performance in many hip and knee applications, its higher crosslink density can, in principle, reduce the polymer's fatigue crack resistance [15]. Consequently, antioxidant stabilization using naturally occurring and biocompatible vitamin E has been proposed and implemented in TJR devices [16]. This approach aims to preserve the wear and oxidation benefits of crosslinking while mitigating the free‐radical‐driven mechanical degradation [17, 18, 19].

As such, vitamin E is ideally suited as a candidate for improving the oxidative resistance to UHMWPE used in mobile bearing TAR implants, without increasing their risk of fatigue cracking/failure. Standard mechanical testing protocols for TAR devices (e.g., ISO 22622) primarily focus on wear evaluations in limited degrees of freedom (e.g., flexion‐extension and axial rotation) and often fail to induce the clinically observed midline fractures. Consequently, the present study pursued two objectives: (1) to establish a multi‐axial loading protocol that reliably reproduces UHMWPE fatigue fractures In Vitro and (2) to examine how oxidation and antioxidant (vitamin E) stabilization influence UHMWPE fatigue performance in mobile bearing TAR implants. It was hypothesized that variations in UHMWPE oxidation would be a critical driver of fatigue life, while varus‐valgus rotation would govern the primary mechanism of fracture. A secondary hypothesis was that vitamin E stabilization would preserve mechanical properties and extend fatigue life, even under oxidizing conditions.

## 2 Methods

The TAR design evaluated in this study is a mobile‐bearing system composed of a cobalt‐chromium (CoCr) tibial tray, a CoCr talar component, and a UHMWPE insert (STAR Ankle, Enovis). Both CoCr components feature a polished surface finish. The talar component includes a rail that corresponds to a trough in the UHMWPE insert, allowing the insert to rotate in the flexion‐extension direction without lateral translation. The tibial tray is flat, allowing for multidirectional translation and axial rotation.

This study evaluated conventional UHMWPE (25 kGy GUR 1020) inserts and vitamin E UHMWPE (75 kGy GUR 1020‐E) inserts (sample sizes for each group/comparison are listed within the bars on each figure and were determined in alignment with industry standards [20]). A subset of each was subjected to accelerated aging for 4 weeks per ASTM F2003‐02 R15 (70°C, 503 kPa, pure Oxygen environment). Aging duration was chosen to generate a near‐critical oxidation state in conventional UHMWPE, as was observed in retrieved fractured mobile bearings. The oxidation index (OI; 0 = undetectable oxidation, increasing values associated with increasing oxidation; 3 = critical loss of mechanical properties) was determined by Fourier‐transform infrared spectroscopy (FTIR) analysis in accordance with ASTM F2102 [21].

**Figure 1:**
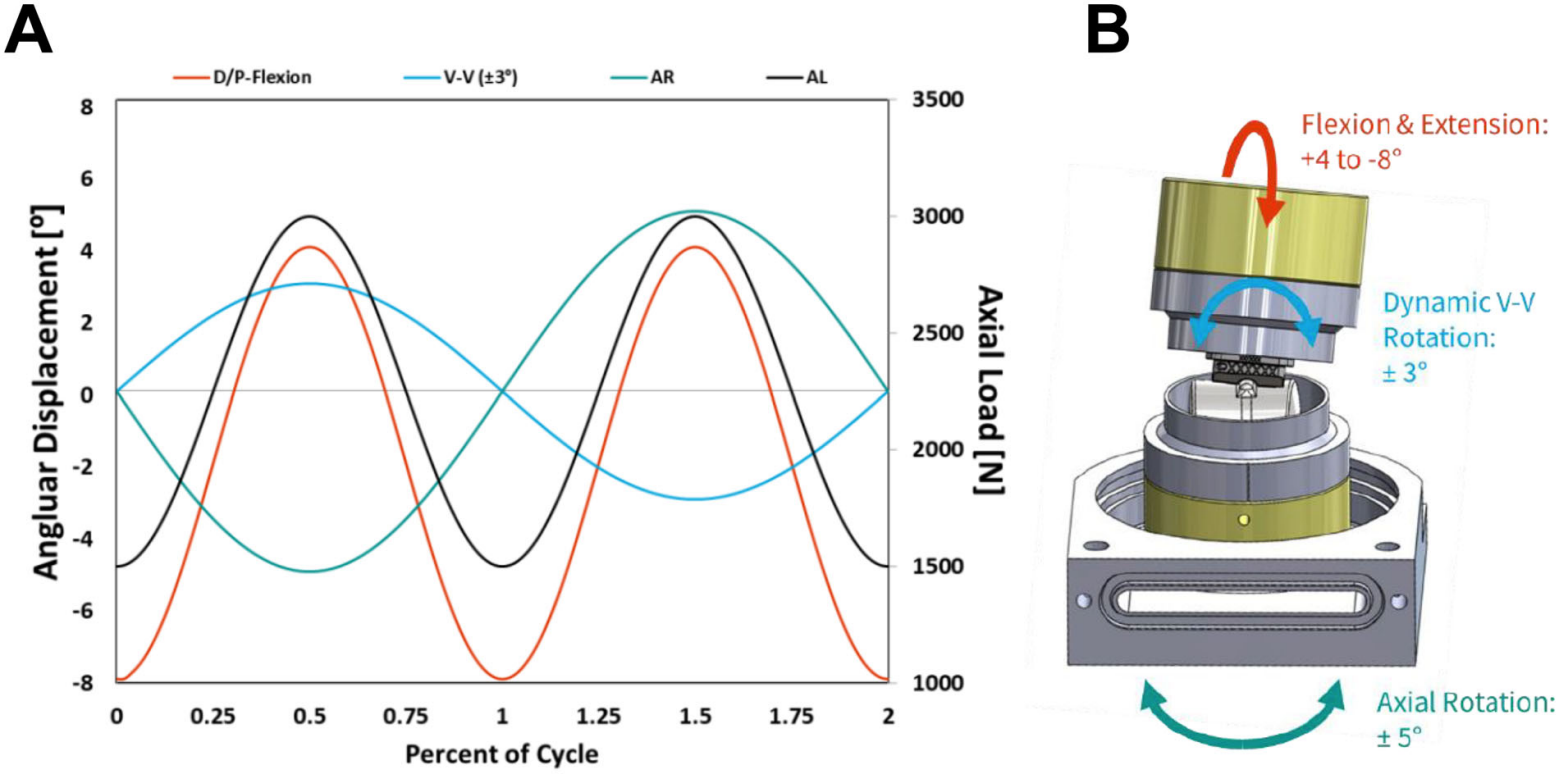
Fatigue testing load waveforms (A) and fixturing (B).

Inserts were subsequently evaluated using a six degree‐of‐freedom servohydraulic wear tester (MTS, Eden Prairie, MN; Figure 1), which applied sinusoidal waveforms for flexion‐extension, axial rotation, varus‐valgus rotation, and axial loading. Samples were submerged in 20 g/L bovine serum at 37 ± 3°C to replicate In Vivo joint conditions. In efforts to generate reliable In Vitro fracturing, two loading protocols were utilized: (1) ± 8° flexion‐extension, ±5° axial rotation, and 0° varus‐valgus angles with cyclic axial loading (*n* = 3 samples; all conventional UHMWPE) and (2) −4/+8° flexion‐extension, ±5° axial rotation, 1500–3000 N axial loading, with ±3° of dynamic varus‐valgus rotation (*n* = 3 samples/group; conventional UHMWPE and vitamin E UHMWPE). These flexion‐extension and axial rotational magnitudes were chosen to align with the ankle wear test standard (ISO 22622:2019[E]), while the dynamic varus‐valgus rotational magnitudes were chosen to represent normal gait [22]. The dynamic force was applied at twice the frequency noted during normal gait to ensure it coincided with the maximum extents of varus‐valgus rotation, to subject the implants to stress conditions greater than those that would occur clinically. Samples in the initial pilot phase without varus‐valgus rotation were run to either fracture or a 1‐million‐cycle runout, oxidized versus non‐oxidized conventional UHMWPE were run to either fracture or a 5‐million‐cycle runout, aged conventional UHMWPE versus aged vitamin E UHMWPE were run to either fracture or a 3‐million‐cycle runout. Fractured specimens were sectioned and examined via scanning electron microscopy (SEM) to visualize fracture surfaces at magnifications up to 200×, enabling identification of crack initiation sites, beach marks, and any plastic deformation indicative of cyclic fatigue. In addition, stereo light microscopy was used to document gross features, such as wire marker positions and midline trough delamination, for correlation with the SEM findings. Oxidation index values were compared using the Mann–Whitney test. Fatigue testing data were analyzed using a *χ* ^2^ test with failed/not‐failed as categorical parameters.

## 3 Results

**Figure 2:**
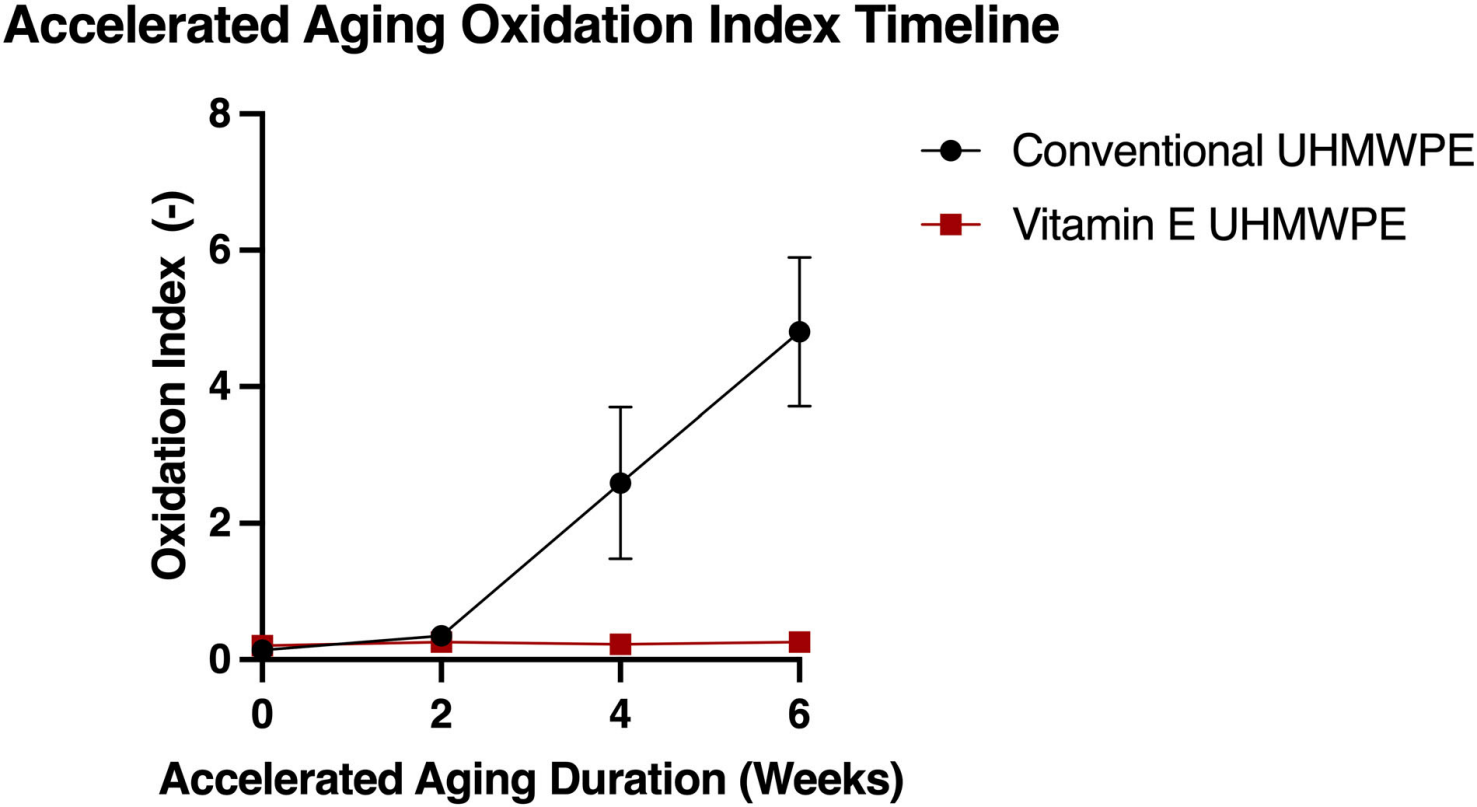
Oxidation index of conventional UHMWPE (25 kGy GUR 1020) and Vitamin E UHMWPE (75 kGy GUR 1020‐E) at various accelerated aging durations.

**Figure 3:**
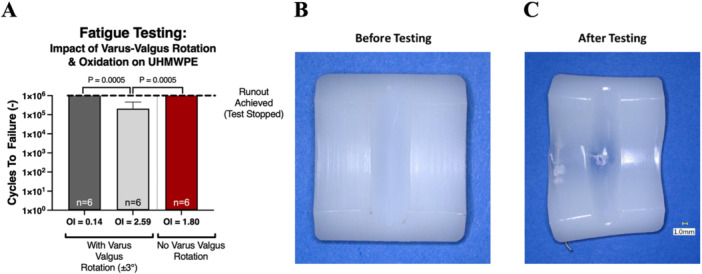
(A) Cycles to failure for oxidized and non‐oxidized UHMWPE (25 kGy GUR 1020) samples with and without ±3° varus‐valgus rotation. Photographs of samples (B) before and (C) after testing without varus‐valgus rotation. Of note is that samples did not exhibit the clinically relevant midline trough failures noted in clinically retrieved samples examined in this study.

Accelerated aging revealed Vitamin E UHMWPE resisted oxidation after 4 weeks of accelerated aging (OI 0 weeks = 0.20 ± 0.05, 6 weeks = 0.26 ± 0.04), whereas the conventional UHMWPE exhibited critical oxidation after that same period (OI 0 weeks = 0.14 ± 0.04, 6 weeks = 4.80 ± 1.09, Figure 2). The first fatigue testing protocol, which excluded varus‐valgus rotation, was unable to generate fractures in near‐critically oxidized (OI 1.80 ± 0.40) UHMWPE (25 kGy GUR 1020) samples up to 1 million cycles (Figure 3A); however, some plastic deformation was noted in the specimen trough region (Figure 3B,C).

**Figure 4:**
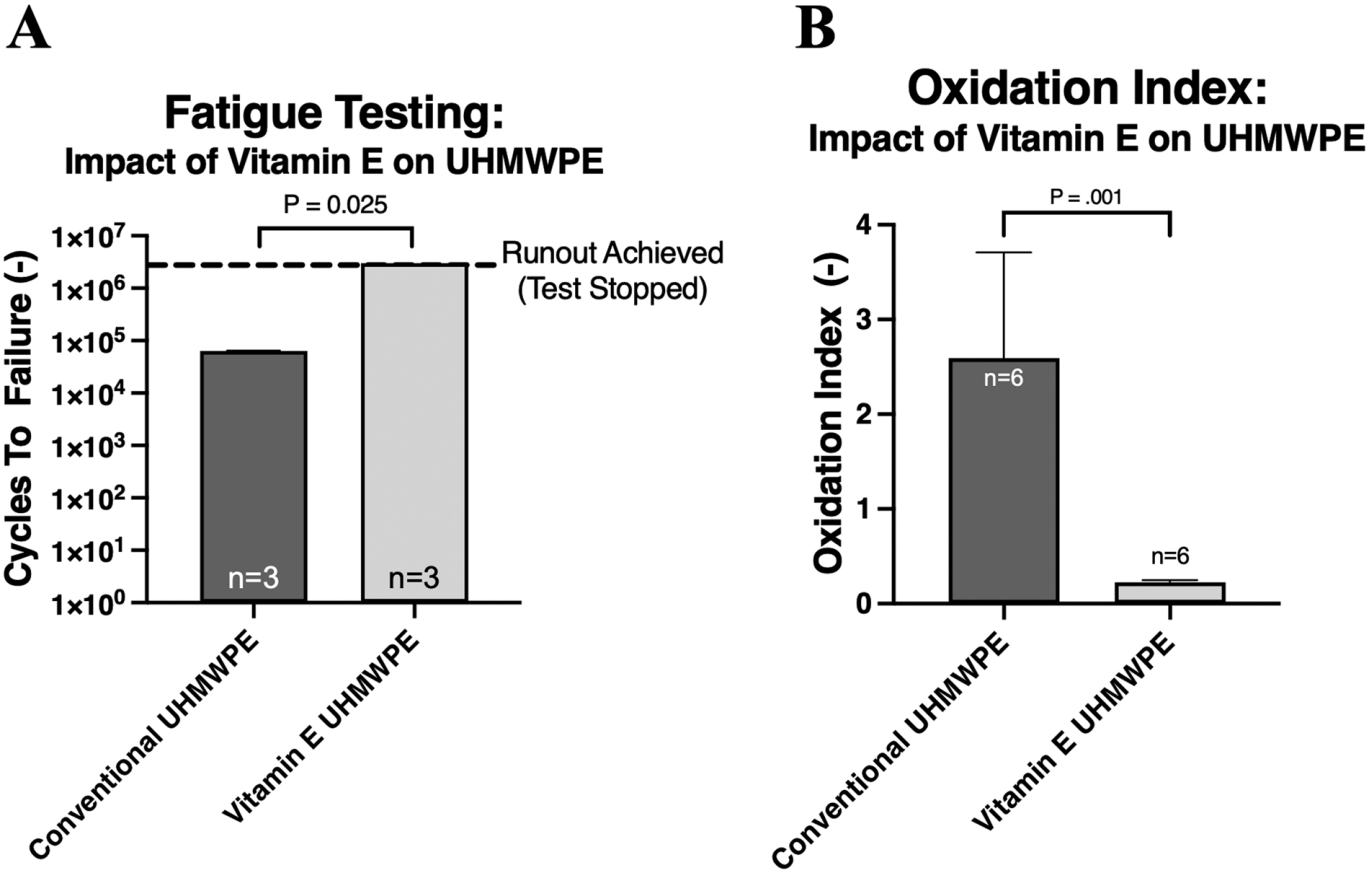
(A) Cycles to failure of 4‐week aged conventional UHMWPE (25 kGy GUR 1020) and 4‐week aged vitamin E UHMWPE (75 kGy GUR 1020‐E). (B) Oxidation index of 4‐week aged conventional UHMWPE (25 kGy GUR 1020) and 4‐week aged vitamin E UHMWPE (75 kGy GUR 1020‐E).

**Figure 5:**
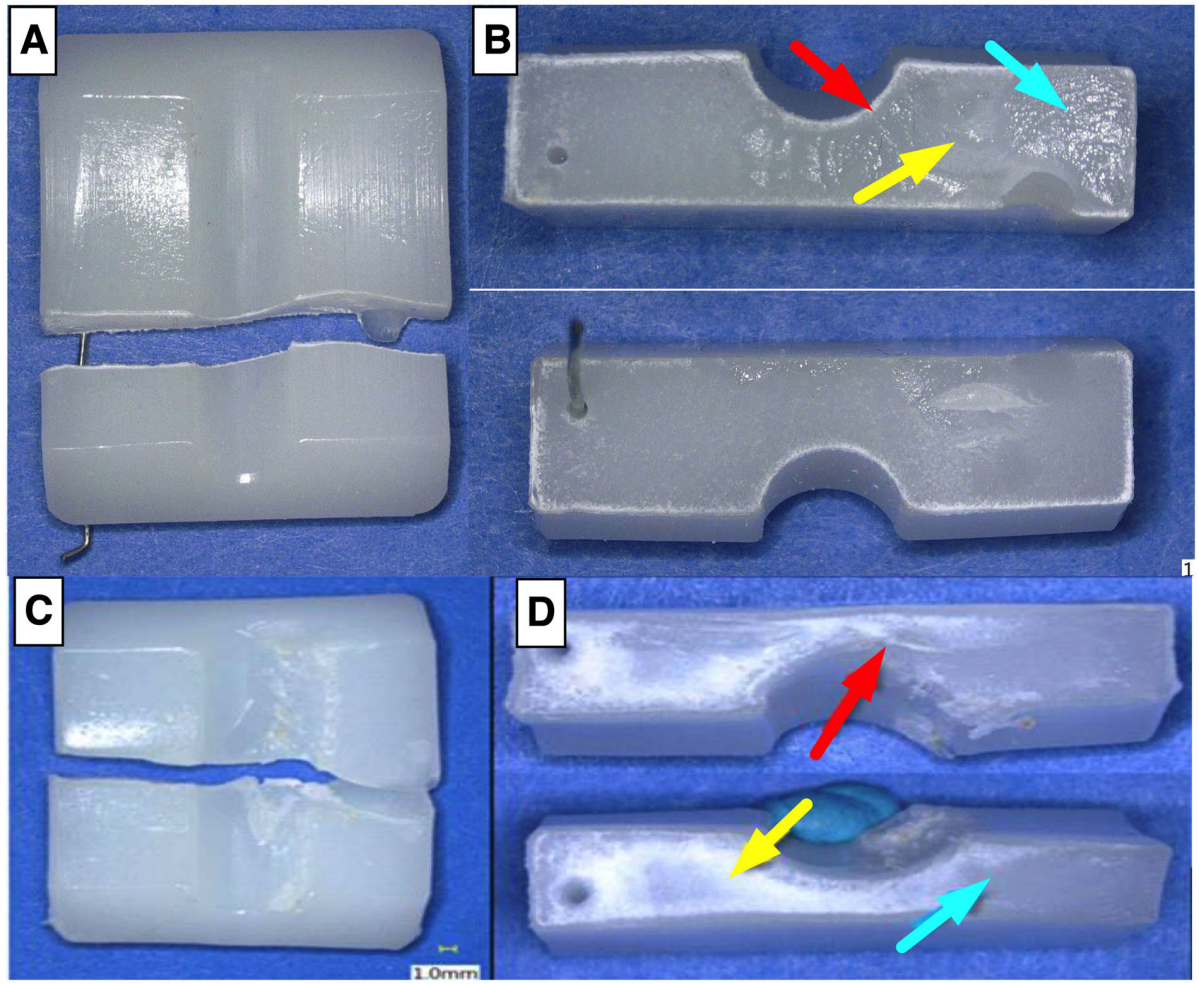
(A and B) Conventional aged UHMWPE insert fracture from the second testing protocol. (C and D) Conventional UHMWPE insert retrieved from a surgical case. Red arrow indicates beach marks/fracture initiation, the yellow arrow indicates ductile deformation, and the blue arrow indicates fast fracture.

The second fatigue testing protocol, which included varus‐valgus rotation, was able to generate fatigue fractures in all near critically oxidized (OI 2.59 ± 1.11) conventional UHMWPE (25 kGy GUR 1020) samples (cycles to failure: mean = 208.7k, std. dev. = 249.3, min. = 45k, max. = 620k) but not in the non‐oxidized (OI 0.14 ± 0.04) conventional UHMWPE (25 kGy GUR 1020) samples (cycles to failure: all samples hit runout at 5MC, Figure 3A, *p* = 0.014). Fatigue testing revealed a significant reduction in cycles to failure in the 4‐week aged conventional UHMWPE (25 kGy GUR 1020) samples (cycles to failure: mean = 63.7k, std. dev. = 1.0k, min. = 62.6k, max. = 64.5k) as compared to the 4‐week aged vitamin E UHMWPE (75 kGy GUR 1020‐E) samples, which did not fail through the 3‐million cycle runout (Figure 4A, *p* = 0.014). The vitamin E UHMWPE samples exhibited significantly decreased oxidation indices after 4 weeks of accelerated aging as compared to the conventional UHMWPE (25 kGy GUR 1020) samples (Figure 4B, *p* = 0.001). Fractography performed on the broken inserts suggests that fractures initiate primarily at the midline of the trough and wire marker, then propagate due to cyclic loading (Figure 5).

## 4 Discussion

These data highlight the well‐known detrimental effects of oxidation on UHMWPE fatigue resistance. Specifically, these data illustrate a 23.9× decrease in fatigue lifespan of UHMWPE inserts at near critical oxidation state (OI = 2.6) as compared to those with little/no oxidation (OI = 0.1, Figure 3A). Similar decreases in implant lifespan with oxidation have been reported previously [12, 23, 24]. As oxidation is a well‐known detriment to UHMWPE, care should be taken to store medical devices containing UHMWPE in nonoxidizing environments (i.e., non‐oxygen permeable foil packaging) to prevent oxidation before implantation. However, it has been proven that conditions within joints produce oxidation in UHMWPE materials [24]. Therefore, it is necessary to minimize In Vivo implant oxidation to achieve maximum implant lifespan.

This study highlights the critical importance of incorporating varus‐valgus rotation in fatigue testing of mobile‐bearing TAR implants to replicate clinically relevant failure modes. Although the second protocol employed a less severe flexion‐extension range (−4° dorsiflexion to +8° plantarflexion, compared to the symmetric ±8° range used previously), it was under this modified protocol that featured varus‐valgus rotation that midline fractures were consistently reproduced. Every conventional UHMWPE sample consistently fractured at the midline, mirroring the morphology observed in clinical retrievals, whereas all vitamin E‐stabilized UHMWPE samples achieved the 3MC runout without failure. These outcomes underscore both the decisive role of rotational stresses (caused by loading during varus‐valgus angulation) in polyethylene fatigue and the protective effect of antioxidant stabilization. Overall, the findings emphasize that rigorous simulation of In Vivo loading is essential to accurately evaluate implant performance and guide future TAR design improvements.

While vitamin E is known for its antioxidant effect on UHMWPE and has been successfully implemented clinically in total hip and total knee applications [17, 25, 26, 27], its impact on TAR devices has not been previously studied. Our data highlight the significant effectiveness of vitamin E at increasing the mobile bearing TAR implant fatigue lifespan. Specifically, these data revealed a 47.2× increase in cycles to failure (including varus‐valgus rotation) in the vitamin E UHMWPE samples as compared to the conventional UHMWPE samples. Over the 4‐week accelerated aging period, the vitamin E‐stabilized inserts exhibited a minimal change in oxidation index (0.20–0.26), whereas the conventional UHMWPE inserts increased dramatically from 0.14 to 4.80, demonstrating a markedly greater susceptibility to oxidation under the same conditions.

These results mirror the improved oxidation resistance and fatigue performance reported in highly crosslinked vitamin E‐stabilized UHMWPE for total hip and total knee arthroplasty [17, 24, 25, 26, 27]. In those larger‐joint contexts, vitamin E effectively quenched free radicals and slowed or prevented oxidative degradation, ultimately reducing wear debris and enhancing implant longevity. The present study indicates that these benefits extend to a mobile‐bearing ankle system as well, despite the distinct geometry and multi‐axial loading environment. While further clinical evaluations are necessary to confirm the magnitude of these improvements In Vivo, our findings suggest the “same story” seen in hip and knee arthroplasty may hold true for TAR, namely that vitamin E incorporation into UHMWPE enhances oxidative stability and prolongs fatigue life—particularly under the coronal‐plane stresses relevant to ankle devices.

Additionally, these data highlight the necessity of including all rotational degrees of freedom (i.e., varus‐valgus rotation) during fatigue testing of TAR devices to generate clinically relevant failures. As demonstrated by the first fatigue testing protocol, even near critically oxidized conventional UHMWPE samples achieved 2 million cycles fatigue test runout without fracture when varus‐valgus rotation was held at zero. When varus‐valgus rotation was prescribed as a sinusoidal displacement profile, insert fatigue life dropped by an order of magnitude, and fractography evaluations revealed similar failure modality as compared to clinically retrieved specimens (i.e., midline fracture, fracture initiation at trough, and ductile deformation before full fracture). Although this study does not replicate a constant varus‐valgus misalignment, prior finite element analyses revealed that the insert trough is in a state of high stress during varus‐valgus misalignment [15]. In our protocol, repeated cycling through these higher angles, rather than maintaining a fixed offset, produced midline fractures—mirroring the localized stress concentrations predicted in static simulations. This outcome highlights that even transient varus‐valgus rotation can negatively impact insert fatigue lift, reinforcing the need for multi‐axial testing conditions that account for clinically relevant coronal plane excursions. As such, manufacturers of TAR devices should implement testing protocols that impart varus‐valgus rotation when designing and assessing implant fatigue life.

There are limitations to this study. Although the testing produced midline fractures, the frequency of larger‐than‐normal varus‐valgus excursions during daily life is not well‐defined, and the loading regimen used in the second protocol may not fully replicate patient‐specific conditions In Vivo. Moreover, although this accelerated aging protocol yielded a near‐critical oxidation state comparable to retrieved samples, it does not definitively correlate to a specific In Vivo duration, limiting direct extrapolation of these findings to long‐term clinical performance. Also, varus‐valgus rotation does not necessarily replicate varus‐valgus alignment of a device. As such, the results presented here should be confirmed with clinical evidence.

## 5 Conclusion

In conclusion, these data illustrate the marked decrease in mechanical properties of TAR inserts when the UHMWPE is heavily oxidized and the effectiveness of vitamin E as an antioxidant to UHMWPE. While these improvements to oxidation and fatigue resistance gained by the addition of vitamin E to the UHMWPE in TAR inserts are encouraging, future research should validate these changes in an In Vivo setting. Additionally, these data highlight the necessity of varus‐valgus rotation in fatigue testing to generate clinically relevant TAR device failures/fractures. Future device development efforts should consider the impact of varus‐valgus loading on device stress and fatigue life.

## Author Contributions

Ruth Heckler, David Safranski, and Ariel Palanca were responsible for funding and conceptualization of this study. Ryan Siskey and Patrick Hall provided strategic input on project design and completed experimental work. James Johnson completed analysis, completed primary draft of manuscript, and contextualized work in current scientific literature. All authors have read and approved the final submitted manuscript.

## Conflicts of Interest

R.H., D.S., and J.J. are paid employees of Enovis. A.P. is a paid consultant of Enovis. The authors have no other conflicts of interest.

## Data Availability

Data are available upon reasonable request to the corresponding author.
